# Plasma biomarkers are stable for an extended period at –20°C: Implications for resource‐constrained environments

**DOI:** 10.1002/alz.087526

**Published:** 2025-01-09

**Authors:** Biniyam A. Ayele, Patrice L. Whitehead, Tianjie Gu, Jamie Arvizu, Charles G. Golightly, Larry D. Adams, Margaret A Pericak‐Vance, Jeffery M. Vance, Anthony J. Griswold

**Affiliations:** ^1^ John P. Hussman Institute for Human Genomics, University of Miami Miller School of Medicine, Miami, FL, USA, Miami, FL USA; ^2^ Addis Ababa University, Addis Ababa, Addis Ababa Ethiopia; ^3^ John P. Hussman Institute for Human Genomics, Miami, FL USA; ^4^ John P. Hussman Institute for Human Genomics, University of Miami Miller School of Medicine, Miami, FL USA; ^5^ Dr. John T. Macdonald Foundation Department of Human Genetics, University of Miami Miller School of Medicine, Miami, FL USA

## Abstract

**Background:**

The Recruitment and Retention for Alzheimer’s Disease Diversity Cohorts in the Alzheimer’s Disease Sequencing Project (REAAD‐ADSP) aims to explore ADRD in diverse ancestral groups within the US and nine countries from sub‐Saharan Africa. While most laboratories store plasma samples ‐80°C for biomarkers, no studies at –20°C have been reported past 2 weeks. Only two sites in Africa possess ‐80°C freezers. As biomarkers can be frozen and thawed, we hypothesized they would be stable at –20°C. The present study was designed to investigate the stability of AD plasma biomarkers at –20°C over time to facilitate studies of plasma biomarkers in resource‐constrained regions.

**Methods:**

Peripheral blood was collected from six anonymous volunteers. Plasma was isolated via centrifugation and 200mL aliquots of plasma were made. Two aliquots per participant were stored immediately at ‐80°C and others at ‐20°C for two, four, six and fifteen weeks then moved to ‐80°C. Plasma biomarkers, pTau181 and Aβ42, Aβ40, NFL, and GFAP, were measured for each time point. Each aliquot was run in duplicate in the assay plate and two aliquots per participant per time point were assayed.

**Results:**

There was a range values at baseline for each biomarker across the six participants: pTau181: 1.6‐3.0pg/mL, Aβ42: 3.7‐6.3pg/mL, Aβ40: 56.2‐106.6pg/mL, NFL: 3.5‐14.9pg/mL, GFAP: 42.9‐71.1pg/mL. At week fifteen, the average change in pTau181 was 0.14pg/mL, less than a 10% overall change. The Aβ42/40 ratio showed a decrease of 3.6% in week 15 compared to the baseline. Variability in NFL and GFAP were higher with increases in all subjects in week 15. Such run variance has been noted for these biomarkers by the manufacturer, thus our results suggest that after fifteen weeks at ‐20°C the assays are performing within specifications.

**Conclusion:**

Up to 15 weeks of storage of plasma at ‐20°C does not significantly change AD related plasma biomarker concentrations beyond assay specifications, though there are slight increases in GFAP and NFL. This suggests that locations lacking ‐80°C storage can utilize ‐20°C equipment and still maintain biomarker fidelity, allowing for greater representation of biomarker studies from low‐ and middle‐ income countries, increasing the diversity of studies in AD.

